# Adjuvant interferon in the treatment of melanoma

**DOI:** 10.1054/bjoc.1999.1225

**Published:** 2000-04-27

**Authors:** J M Kirkwood

**Affiliations:** Department of Medicine, University of Pittsburgh Medical Center and University of Pittsburgh Cancer Institute, Pittsburgh, PA, 15213–2582, USA


British Journal of Cancer (2000) 82(10), 1755–1758
© 2000 Cancer Research Campaign
DOI: 10.1054/ bjoc.1999.1225, available online at http://www.idealibrary.com on IDEAL®

Letters to the Editor

### Adjuvant interferon in the treatment of melanoma

**Sir**

We write in response to the editorial by MR Middleton regarding the first analysis of the intergroup E1690 trial of high- and low-dose interferon for high-risk melanoma conducted in the US between 1990 and 1995. This large trial accrued 642 patients with resectable deep primaries or node-positive disease to a three-arm trial of observation, high-dose interferon α-2b (IFN-α-2b) for 1 year or low-dose IFN-α-2b for 2 years. The editorial was submitted in February, after presentation of this trial's preliminary analysis to the ESMO in late 1998. But this study has now been presented in much greater detail to the American Society of Clinical Oncology, and is in review for publication. Middleton's editorial concluded that the high-dose regimen can not be recommended as the standard of therapy for high-risk melanoma patients; however, this conclusion is based on very incomplete information, since the data from E1690 have yet to be fully presented in a formal publication. We would like to correct a number of errors in the editorial, and urge the community to carefully consider the data of the formal E1690 trial when they are published this year.

1. In reference to differences in overall survival between the study population in the E1690 trial and the study population in the preceding E1684 trial, Dr Middleton writes, 'This unexpected difference in the results of the two studies can be explained by an improvement in the outcome of these patients kept under observation … [and] may be due to changes in the staging and surgical techniques: for example, sentinel – node mapping…'.

**Response**: The E1690 trial results differ most significantly from E1684 in regard to post-relapse survival of patients in the observation arm, and this has nothing to do with differences in staging or surgical technique. In point of fact, very few patients on E1690 were staged using sentinel lymph node biopsy, and none were assessed as node-positive based on immunochemical or reverse-transcriptase polymerase chain reaction analyses.

2. Dr Middleton also writes, 'In the earlier trial, significant differences in overall survival were only seen amongst patients with clinically apparent lymphadenopathy prior to resection.'

**Response**: Significant differences in overall survival in E1684 were seen in the intent-to-treat analysis of the *entire* population. These differences, which were statistically significant for the entire trial, were yet more significant in the histologically node-positive population, which comprised 89% of the accrual to E1684. In point of fact, the hazard ratio associated with high-dose IFN-α-2 therapy on E1684 was greatest (indicating the most improvement in survival) for patients with clinically negative but pathologically positive nodes.

We would agree that the lack of efficacy of low-dose IFN-α observed in the E1690 trial, coupled with the negative results of the WHO 16 trial of low-dose adjuvant IFN lead to a compelling conclusion that low-dose IFN-α-2b is less effective than the high-dose regimen. We would also agree that 'there seems little doubt that high-dose interferon has an impact on melanoma, and can delay the time to relapse in high risk melanoma patients.'

We believe that the consistent improvement in the continuous relapse-free survival of high-risk melanoma patients receiving high-dose IFN-α-2b seen in both E1690 and E1684 corroborates the biologic activity of this regimen. With regard to the survival benefit observed in E1684, this trial was conducted at a time when IFN-α was not available for cross-over therapy, and all patients treated on E1684 had undergone full lymphadenectomy and so had no opportunity for cross-over. This situation is distinctly at variance with E1690. In E1690, there was more than a twofold larger number of patients with clinically negative but unresected lymphatics, and these patients had the opportunity to cross-over to IFN-α therapy if they had a regional nodal recurrence. In fact, they did so in substantial numbers, asymmetrically pursuing IFN salvage therapy from the observation arm after failure in regional lymphatics. It is well recognized that regional lymph node relapse is the most frequent site of relapse in melanoma patients who have not undergone lymphadenectomy, and this occurrence in more than 40% of the total number of patients treated on E1690, provided an opportunity for a confounding second exposure to IFN at relapse. Such was not the case in E1684. A retrospective analysis of salvage therapy demonstrated significantly greater numbers of patients from the observation arm than from the high-dose IFN arm were treated with IFN-α salvage therapy. This provides a plausible explanation for the differences between the E1684 and E1690 trials in terms of post-relapse and overall survival outcome.

We would urge the readership to review the data from E1690 when published in *J Clin Oncology* and to draw their own conclusions. It is our responsibility as oncologists to present the data regarding high-dose IFN-α-2b to our high-risk melanoma patients fully and in a balanced fashion. Ultimately, it should be the patient's choice to accept or reject treatment.

*JM Kirkwood,*
*Department of Medicine,*
*University of Pittsburgh Medical Center and*
*University of Pittsburgh Cancer Institute,*
*Pittsburgh, PA 15213–2582, USA*

DOI: 10.1054/ bjoc.1999.1244, available online at http://www.idealibrary.com on IDEAL®

### Adjuvant interferon in the treatment of melanoma – reply

**Sir**

Although accepted for publication in February 1999 the editorial in question was amended in proof to take into account Dr Kirkwood's presentation to the ASCO congress, as is made clear

